# Memory for Lectures: How Lecture Format Impacts the Learning Experience

**DOI:** 10.1371/journal.pone.0141587

**Published:** 2015-11-11

**Authors:** Trish L. Varao-Sousa, Alan Kingstone

**Affiliations:** Department of Psychology, University of British Columbia, Vancouver, British Columbia, Canada; University of Westminster, UNITED KINGDOM

## Abstract

The present study investigated what impact the presentation style of a classroom lecture has on memory, mind wandering, and the subjective factors of interest and motivation. We examined if having a professor lecturing live versus on video alters the learning experience of the students in the classroom. During the lectures, students were asked to report mind wandering and later complete a memory test. The lecture format was manipulated such that all the students received two lectures, one live and one a pre-recorded video. Results indicate that lecture format affected memory performance but not mind wandering, with enhanced memory in the live lectures. Additionally, students reported greater interest and motivation in the live lectures. Given that a single change to the classroom environment, professor presence, impacted memory performance, as well as motivation and interest, the present results have several key implications for technology-based integrations into higher education classrooms.

## Introduction

The way that educational information is conveyed has changed greatly in recent decades. One major change has been in the increase in courses offered online or through distance education. For example, Allen and Seaman [1] report that in the United States over a quarter of college students take one or more online courses. Investigations of how different educational settings may impact cognitive factors, such as memory, often involves comparing groups who are offered the material in various ways, for example live versus online “podcast” versions [2–5]. Unfortunately, comparing a live classroom to a podcast version of the same lecture involves a manipulation of not just material modality and presentation but also the physical setting, social environment, control of material (e.g., the ability to replay the lecture), and so on. Without controlling or equating these other factors the reason for a change in performance, or lack-there-of, cannot be isolated. This lack of thorough investigation is noteworthy given that, in lieu of traditional classrooms, many universities offer online courses, blended-learning, or synchronous learning environments (where students watch a live video stream of the lecture while at a remote-location), with the intention that these changes are providing a beneficial change to the educational experience.

The present study investigated whether memory and mind wandering differ between a traditional classroom and a classroom where the lecture is presented via pre-recorded video. By manipulating only the "live" quality of the professor and maintaining all other aspects of the classroom setting (e.g., scheduled time and location, presence of peers, etc.) we can begin to isolate what aspects of the lecture, live versus video format, impact the learning experience. In the following pages we highlight the technological shifts in educational settings that have recently taken place, and review how these relate to past investigations of memory and mind wandering (as a measure of inattention) in classroom settings.

### The shift to technology-based learning

Advances in technology have allowed for learning in various locations and modalities (e.g., watching a video-recording of a lecture on a bus, or participating in an online course taught on the other side of the world). The growth of non-traditional classroom-based learning is demonstrated by the breadth of the courses and programs offered online. At Canadian universities up to 20% of all students enroll in online courses [6]. And presently, many universities have implemented an approach of blended-learning, which combines traditional classroom teaching with indirect teaching (e.g., teleconferencing and requiring personal research outside of class time to better facilitate in-class discussion, etc.). Indeed, given the ease that one can enroll in courses outside of one’s current city, or even country, many choose to take part in online learning for reasons of accessibility and time flexibility (e.g., [7] for review). The newest educational trend, Massive Open Online Courses (MOOCs), has exemplified the desire for continued education.

As hundreds of thousands of students from around the world sign up for online courses there has arisen the opportunity for large quantities of data to be collected—these data have focused on lecture length, drop-out rates, accessibility, participation, overall sense of satisfaction and interest [8–10]. But as of yet there has been no systematic investigation of the real-time cognitive experience and learning impact of video versus live lecture environments. While the opportunity for accessible and continuous learning is unquestionably important, key questions have been raised: how do different learning environments impact the learning experience? Specifically, are there cognitive costs or benefits to changing the live lecture experience? And what is the subjective student experience of different learning environments? Answering these questions requires a detailed investigation of what happens when the classroom changes.

### Do changes to the classroom impact cognitive factors?

To gain an understanding of how different learning environments impact the learning experience, and whether there are cognitive costs or benefits when the classroom setting is changed, we must first consider which cognitive factors are likely to be impacted. To date, researchers have focused largely on memory performance when comparing classroom environments. However, studies have yielded mixed outcomes ranging from: equivalent performance in technology-based learning and live classrooms [2–4], to benefits for e-learning [11], to benefits for classroom learning [12,13]. As we note in the next section, methodological and/or situational differences within and between studies has varied dramatically, making it unclear what, if, and how specific factors are responsible for the different memory effects. In contrast, our study strives to hold all variables constant, save one—the nature of the instructor's presence.

Another factor that may be impacted by changes to the classroom environment is attention, a cognitive process that is often measured with regard to mind wandering reports. Indeed, a daily diary study found educational settings to be the most common location for mind wandering to occur [14], which is convergent with reports that mind wandering in classrooms occurs 30–40% of the time [15–17]. While these studies provide information on how attention may lapse during a live lecture setting, no studies to date have investigated whether the amount of mind wandering changes as a function of classroom setting. What limited research has been conducted in classroom styled environments suggests there is a negative correlation between mind wandering reports and memory performance [16,18–20]. Given that mind wandering can negatively impact memory performance we reasoned that both factors should be investigated when considering a change in an alternative learning environment.

### The present study

To date, fundamental methodological differences between learning environments prevent a straightforward comparison between the performance effects of live and online situations. For instance, students in e-lecture environments can re-watch material and are often encouraged to do so [3,11]. Additionally, the environment where learning takes place is often vastly different (e.g., professor presence, material modality, social setting, location of viewing, etc.) making it difficult to pinpoint what factor, or combination of factors, translates into differences in performance. By systematically manipulating individual factors of the learning environment one can begin to isolate what cognitive changes, if any, emerge in different settings. The present investigation manipulated only one factor, physical professor presence, while holding all else constant, allowing for a direct comparison between learning environments.

We believe that investigating the cognitive impact of live professor presence is a unique and critical first manipulation to the classroom setting. Studies on the impact of social presence have indicated a cognitive and behavioural impact of feeling as though one is being watched [21–23] and that the possibility for social interaction impacts behaviour [24–26]. A live classroom setting employs both these social presence factors: the professor being physically present might impact behaviour (e.g., whether a text message is sent, how focus of attention is maintained) and there is also the potential for interaction (e.g., being asked to answer a question). Furthermore, we believe that the instructor presence is perhaps the most critical change between live and online settings. Beyond implications of the effects on human behaviour listed above, the role of professors as instructors is a crucial part of the university objective. Institutions are ranked for their teaching capabilities and many instructors must carefully balance their time between teaching, research and service. Determining whether professor presence impacts the learning experience is an important question for university initiatives. On the one hand, professors are typically required to perform teaching duties, and must consider the value they impart on the teaching experience by being physically present. On the other hand, pre-recording lectures could free up professor resources allowing for more one-on-one time with students.

By manipulating only live professor presence we maintain all other factors that might vary between a live and online setting: the social presence of peers, consistent pacing (e.g., not stopping the task and re-starting at a later time), physical environment (e.g., lecture hall) and any other factors that might differ between settings. This means that any effects found can be attributed to the single manipulation: live professor presence. One additional benefit of the present study’s design is that it is more ecologically valid than traditional lab-based studies [27]. That is, the participants are students enrolled in the Introductory Psychology class where the study takes place and the materials being presented are lectures in the course that deliver information relevant to the students successfully passing the course. This stands in sharp contrast to testing students individually in a laboratory on information that has little if anything specific to do with any course they are taking, and where motivation for taking part may be supplemental participation credit or some nominal form of remuneration.

## Method

Ethics approval for this study was obtained from the University of British Columbia Behavioural Research Ethics Board. Written, informed consent was obtained from participants.

### Participants

An invitation to participate in the study was extended to students registered in two sections of Psychology 102: Introduction to Developmental, Social, Personality, and Clinical Psychology at the University of British Columbia. A total of 276 students (180 female; *M*_*age*_ = 19.84 years, *SD* = 3.41) participated in both sessions of the study in return for course credit. An additional 82 participants (Live only = 37; Video only = 45) did not complete both sessions of the experiment and so were excluded from analyses (with one notable exception which we flag for attention in the Results section).

### Materials and Measures

The key areas of interest in the present study were memory and mind wandering reports. Additionally, we measured interest and motivation to investigate whether these ratings differed based on the learning environment, and whether these factors correlated with memory or mind wandering in classroom settings. At the beginning of each session students who wished to participate in the study were provided a response sheet, this sheet was used to record all participant information and dependent variable responses.

#### Lecture Materials

Content was pre-determined by the professor. Two lectures were presented that encompassed the theme of Treatments of Clinical Disorders, but differed in topic: Psychotherapy and Drug Treatment. Session 1 (Live) differed for each class section, such that one class was presented material on Psychotherapy and another received material on Drug Treatment. These lectures were video-recorded by a researcher and then presented for the second session of the experiment (Video version). This allowed us to change the topic for Session 2 (Video) so that students did not see a repeat of the same topic. Each lecture was approximately 60 minutes in length. Lecture material was supplemented with PowerPoint slides.

The order of conditions led to an unbalanced design however there are practical, conceptual, and statistical reasons for. First, a practical consideration: the professor needed to present the material so that we could record it and we did not want to tamper with her presentation style in any way that could negatively impact the ecological validity of the study or quality of the Live versus Video comparison (e.g., have her pre-record the lectures in her office or in front of an empty classroom). Therefore, we recorded the Live lectures that came immediately before the Video lecture presentations, thereby minimizing any other differences between conditions, while counterbalancing the lecture topic. A conceptual consideration is that, in actuality, the live lecture condition is just one of many live lecture experiences that the students have had, and will continue to receive, in their educational experiences, and thus its occurrence is not uniquely defined with regard to the Video condition, i.e., as coming before or after the video condition. Finally, we reasoned that if there was any effect of order, placing the Video lecture second would, if anything, operate *against* our finding an advantage for the Live lecture relative to the Video lecture, i.e., it represented a conservative test of the working hypothesis that Live lectures enhance student performance. In other words, student performance in the Live lecture would suffer any cost of being first, and the Video lecture any benefit of being second (e.g., the students in the Video condition could expect to be tested on the material that occurred at the time that they were probed for mind wandering). As we report in the results, our intuitions were validated and our discovery of a Live lecture advantage may be a conservative estimate of the actual difference between Live and Video lectures.

#### Mind Wandering Probes

Over the course of each lecture 6 mind wandering probes were displayed on lecture slides. The timing of these slides was predetermined, such that probes occurred just after the presentation of lecture material that would later be part of the memory test. The slide presented the question “In the moment prior to this slide, were you mind wandering?” (based on [16], p. 161) and students were asked to circle “Yes” or “No” on the response sheet provided. Participants were given an average of 24 seconds to record their responses.

#### Memory Test

To identify differences in retention between Live and Video lecture settings a 6 item True-False memory test was given at the end of each lecture (see S1 Appendix for questions). Memory test questions were presented on a PowerPoint slide at the end of the lecture and responses were recorded on the participant response sheet. Students were asked to work independently and not use their notes as aid to answer the questions.

#### Interest Rating

To investigate whether ratings of interest differed between the two learning environments, participants were asked to rate their interest in the lecture topic on a 5 point scale at the end of each session (where 1 = very little interest and 5 = high interest).

#### Motivation Ratings

To investigate whether motivation to remain attentive differed between the two lecture settings a 5 point scale was created and included on the participant response sheet (where 1 = very unmotivated and 5 = very motivated). To further compare motivation across the lectures, at the second session (Video lecture) students were also asked to rate which version they were most motivated to attend to: Live, Video or Equally Motivated.

### Procedure

Participants completed the experiment during their regularly scheduled class, across two class sessions. Prior to the study date a researcher attended the class and explained the broad nature of the study to the students and, critically, that they were under no obligation to participate in the study, nor would the decision to not participate have any impact on their course grade. The day of each lecture, students were provided separate informed consent and response sheets and given instructions for the experiment by the researcher. The instructor did not provide any information about the experiment to the students. Students were asked to provide basic demographic information on the participant response sheet. Students were provided a definition of mind wandering: “Any thoughts that are experienced that are not related to the material being presented” and examples were provided (e.g., thoughts about lunch; thoughts about past weekend events; concerns about coursework, etc.). There were no differences in instruction provided to the two sessions. Upon completion of the class participants turned in the consent form and response sheet to the researcher.

## Results

We counterbalanced across lecture topic as it was not a variable of interest in our study. Nevertheless, we conducted a mixed effect model analysis with topic as a factor to examine if it might have a differential impact on our data. This analysis revealed that topic was a significant factor for mind wandering reports, but not for any other variable. There were no meaningful interactions in the data. This analysis allowed us to be confident that collapsing across the two counterbalanced lecture topics to increase our statistical power would not compromise the results and interpretations of the data.

Memory test performance, Mind wandering reports, Interest and Motivation Ratings were all assessed using paired samples tests, with Lecture Style as the Independent variable. We used an alpha level of .05 for all statistical tests. Table 1 displays a summary of all descriptive statistics. Visual representations of these data are presented as violin plots in S1 Fig.

**Table 1:** Summary table of task means as a function of Lecture Style, with standard deviation in parentheses.

Measure	Live	Video
Memory (%)	74 (20)	70 (21)
Mind wandering (%)	48 (23)	49 (27)
Interest Rating	3.08 (.82)	2.84 (.87)
Motivation Rating	3.63 (1.01)	3.20 (1.16)

### Memory Test

The average Memory accuracy from the Live lecture was 74% (*SD* = 20%) and 70% (*SD* = 21%) during the Video lecture. A paired-samples t-test indicated that participants performed significantly better on the Live lecture memory test compared to the Video lecture memory test, *t*(275) = 2.83, *p =* .005, *Cohen's d* = .17.

Note that in the Methods section it was proposed that our unbalanced design, with Live preceding Video sessions, may actually yield a conservative estimate of the actual difference between Live and Video memory performance. This is because participants in the Video session could use their previous experience in the Live session to correctly anticipate that they would be tested on lecture material that co-occurred with the mind wandering probes. To test this we compared Memory accuracy for the participants who only completed the Video session (*n* = 45, and as such received the Video session “first”) against a randomly selected subset of Video condition participants (*n* = 45) who attended both the Live and Video sessions (and therefore, received the Video condition second). This analysis revealed that those who received the Video condition second performed significantly better (*M* = .71) on the memory test compared to those who received the Video condition “first” (*M* = .61), *t(*88) = 2.2, *p* = .03, 95% CI [.02, .18]. This confirmed our hypothesis, that the enhanced memory performance of those in the Live condition, who received the test material first, relative to the memory performance of those participants who received the Video condition second, is a conservative estimate of the benefit of receiving a Live lecture.

### Mind wandering Reports

Average Mind wandering reports was 48% (*SD* = 23%) during the Live lectures and 49% (*SD* = 27%) during the Video lectures. A paired-samples t-test revealed no significant effect of Lecture Style, *t*(275) = -.81, *p =* .42.

### Interest Rating

Twenty-one students did not complete this section of the response sheet for one or both of the sessions, meaning that their data could not be included for this analysis (*N* = 255). Average Interest rating was 3.08 (*SD* = .82) for the Live lecture and 2.84 (*SD* = .87) for the Video lecture. As Interest rating was reported using an ordinal scale, a non-parametric, Wilcoxon Signed Rank test was completed. This test revealed that Interest Ratings were significantly higher (indicating greater interest) in the Live lecture compared to the Video lecture, *Z* = -4.26, *p* < .001.

### Motivation Ratings

Twenty-one students did not complete this section of the response sheet for one or both of the sessions, meaning that their data could not be included for this analysis (*N* = 255). As per Interest ratings, an ordinal scale was used to measure Motivation rating and so a Wilcoxon signed rank tests was completed. This test revealed that Motivation Ratings were significantly higher (indicating greater motivation) in the Live lecture (*M* = 3.63, *SD* = 1.01) compared to the Video lecture (*M* = 3.20, *SD* = 1.16), *Z* = -5.05, *p* < .001.

The response sheet for the second session (Live condition) included a second motivation question: participants were asked to reflect on both sessions and select which lecture they had been most motivated to attend to. Of the participants who completed this section of the response sheet (*N* = 265), 61% of individuals reported being more motivated to attend the Live lecture, 28% reported equal motivation and only 11% reported they were more motivated to attend to the Video lecture, a chi-squared one-variable test indicated that the ratings were significantly different, *X* ^2^ (2, *N* = 265) = 101.65, *p* < .001. These results are consistent with the Motivation Rating reports made at the end of each session which revealed greater motivation to attend to the Live lecture over the Video lecture.

### Correlational Analyses

Prior work has investigated the impact that interest and motivation have on cognitive factors such as memory, and mind wandering [4,16,28–30]. Unfortunately, most of these studies did not take place in classroom settings (see [4,16] for exceptions). As such, we investigated which, if any, of these correlations extend to an actual classroom setting. Correlations were assessed using a Spearman's rho calculation. Correlation coefficients and respective confidence intervals are displayed in Table 2. Correlation plots for these data are illustrated in S2 Fig.

**Table 2:** Spearman's rho correlations for factors of interest (*N* = 265).

	MW—Live	Interest Rating—Live	Motivation Rating—Live	MW—Video	Interest Rating—Video	Motivation Rating—Video
Memory—Live	-.071	0.15*	.10			
MW—Live		-0.27*	-0.24*			
Memory—Video				-0.16*	0.15*	0.17*
MW—Video					-0.32*	-0.34*

*Note*. MW = mind wandering.

* indicates a correlation within the 95% confidence interval.

The correlation between mind wandering and memory was found to be non-significant for the Live lecture, however was significant for the Video lecture, indicating that increased mind wandering reports were related to decreased memory test performance, *r*_=_ -.16, *p* = .01, 95% CI [-.27, -.04]. Previous research suggests that Interest and Motivation Ratings correlate positively with memory performance, such that greater motivation or interest ratings are related to higher memory performance. All correlations were in this predicted direction, with significant correlations for all but memory and motivation in the Live condition, *ps* < .05. As per Lindquist and McLean (2011), we predicted a negative relationship between mind wandering and interest, where greater Interest ratings would be related to fewer mind wandering reports. Indeed, correlations were significant for both Live and Video sessions: *r*_=_ -.27, *p* < .001, 95% CI [-.38, -.16] and *r*_=_ -.32, *p* < .001, 95% CI [-.42, -.20], respectively. Finally, and as per research in non-classroom domains, there was a negative relationship between mind wandering and motivation. This result suggests that lower motivation ratings are associated with a higher number of mind wandering reports, for both Live and Video sessions: *r*_=_ -.24, *p* < .001, 95% CI [-.35, -.12] and *r*_=_ -.34, *p* < .001, 95% CI [-.44, -.23], respectively.

## General Discussion

The current study investigated how different learning environments (live in-class versus pre-recorded video) impact two aspects of the learning experience: memory performance and mind wandering. We also examined how subjective experiences, specifically interest and motivation, differ between these environments and what impact, if any, they have on memory and mind wandering.

Our finding that memory performance was significantly higher in the Live session compared to the Video session suggests that the acquisition and retention of the lecture information benefitted from having the lecture delivered by a professor who was physically present in the classroom. Moreover, as noted previously, the design of the present study was such that this significant difference is, if anything, an underestimation of the actual magnitude of the Video lecture disadvantage. Perhaps the most obvious and important implication of this finding is that in real world settings, where lecture material is delivered in an online format over video (e.g., MOOCs), students taking such courses may retain much less lecture information than those who receive the material in a classroom with a professor.

Interestingly, the difference in memory performance between Live and Video instruction cannot be attributed to differences in attention vis-à-vis mind wandering as our results indicate that mind wandering was unaffected by the lecture format. That said, the *impact* of mind wandering in the Live versus Video conditions does not appear to be equivalent. The correlation data reveal a negative relationship between mind wandering and memory in the Video condition, such that memory declined as mind wandering increased, but a similar relationship was not observed in the Live condition (see Table 2). This suggests that memory for online material is more sensitive to shifts in attention when the material is delivered online. As discussed in detail below, this may arise because the material is found to be less interesting, and the students are less motivated, when watching a Video lecture than when they receive it Live.

Subjective ratings revealed that students found the material less interesting and were less motivated when the lecture was on video. The change in interest for the material is most critical, as the correlation data indicate that for both Live and Video lectures memory performance changes with a shift in interest, i.e., memory performance declines when interest declines. It is our position that this present effect is a conservative estimate of the real world impact of delivering course material online to students. The reason for this being that a number of factors were present in this design that would not be in a pure online setting, for example: (i) it was a novel experience, (ii) the students received the video information in their usual classroom and normal lecture time, and (iii) students were surrounded by their peers, which offers a well-established social benefit [31–33]. Thus our study suggests that not only may students' retention of course material suffer relative to their in-class cohorts who receive live lectures, but with the decline in interest and motivation, students may well struggle to keep up with a course; a possibility that converges with the high attrition rates for MOOCs: 15–40% of the students enrolling in MOOCs fail to complete them [34–36].

Interestingly, several prestigious institutions such as Stanford University, Harvard University and the University of British Columbia have bridged the gap between a completely traditional class setting and online learning, via blended-learning designs. These settings employ course-credit classroom sessions where part of the learning takes place in a traditional classroom however much (up to at least half) of the learning takes place online, requiring students to watch lectures on their own time and participate in online tasks. This setting combines the "perks" of online learning (e.g., self-pacing, freedom of location) with the traditional learning environment. In light of the present study, one obvious next step would be to monitor students’ access of the online material and measure performance (e.g., memory) and subjective experience for both environments. As the critical benefit of the present study was that it took place in a natural environment, a future more longitudinal investigation where lectures are systematically alternated between live and video presentation, as in blended-learning, would provide the opportunity to extend the present results and further determine the cumulative effects of professor presence (real versus video) on student learning. Similarly, how these effects vary as a function of individual differences in aptitude and learning motivation styles, is also very much an open and interesting question ripe for future investigation.

The present study spearheads a much needed initiative for careful examination of differences in cognitive impact and subjective experience between traditional classroom learning and online learning environments. We recognize that there may be benefits to online learning (e.g., material can be re-watched, students can watch at a more convenient time, etc.) or at least different ways that students can interact with materials compared to with a live lecture [3,10,11]. Whether these benefits outweigh the costs we have reported here is an important issue for future investigation. With growing demand for flexible and convenient online learning settings, the costs of moving forward too quickly and without making amends for cognitive impacts could mean compromising a tried-and-true learning experience. The present study illustrates that students' memory performances differ between live and video versions of a lecture, and that they are more motivated and interested when attending a session with a live professor present. These results indicate that natural settings can be used to investigate the differences in learning behaviours and experiences, and that comparisons need not be limited to laboratory environments.

## Supporting Information

S1 Appendix Memory test Questions. (DOCX)

S1 Fig Violin plots for each of the dependent variables. Violin plots indicate the density distribution each dependent variable. Light coloured plots represent performance in the Live condition and Dark coloured plots represent performance in the Video condition. The white dot represents the median and the black bar represents the interquartile range. (A) Memory performance percentage. (B) Percentage of mind wandering reports. (C) Interest Ratings, where 5 indicated high interest and 1 indicates low interest. (D) Motivation Ratings, where 5 indicated high motivation to attend and 1 indicates low motivation to attend.
(PDF)

S2 Fig Jittered correlational plots for all dependent variables. Jittering was used to distinctly display data points that would otherwise overlap (due to scales not being continuous) and provide a clear visualization of the data. (A) Displays correlations between the variables for the Live condition. (B) Displays correlations between the variables for the Video condition.
(PDF)

## References

[1] Allen IE, Seaman J. Learning on Demand: Online Education in the United States, 2009. Sloan Consort [Internet]. 2009 Dec; Available: http://eric.ed.gov/?id=ED529931. Accessed 2015 Jul 3.

[2] Abdous M, Yoshimura M. Learner outcomes and satisfaction: A comparison of live video-streamed instruction, satellite broadcast instruction, and face-to-face instruction. Comput Educ [Internet]. 2010 9;55(2):733–41. Available: http://linkinghub.elsevier.com/retrieve/pii/S036013151000076X. Accessed 2014 Dec 9.

[3] Demetriadis S, Pombortsis A. e-Lectures for Flexible Learning : a Study on their Learning Efficiency. 2007;10:147–57.

[4] Sankaran SR, Bui T. Impact of Learning Strategies and Motivation on Performance: A Study in Web-Based Instruction. J Instr Psychol [Internet]. 2001 9 1 [];28(3):191 Available: https://www.questia.com/library/journal/1G1-79370574/impact-of-learning-strategies-and-motivation-on-performance. Accessed 2015 Jul 3.

[5] Spickard A, Alrajeh N, Cordray D, Gigante J. Learning About Screening Using an Online or Live Lecture. J Gen Intern Med. 2000;17(7):540–5. 10.1046/j.1525-1497.2002.10731.x PMC1495076 12133144

[6] Canadian Virtual University. Online University Education in Canada : Challenges and Opportunities. 2012;(January).

[7] Luppicini R. Review of computer mediated communication research for education. Instr Sci [Internet]. 2006 7 20;35(2):141–85. Available: http://link.springer.com/10.1007/s11251-006-9001-6. Accessed 2014 Dec 13.

[8] Grainger B. University of London International Programmes: MOOC Report [Internet]. 2013. Available: http://www.londoninternational.ac.uk/sites/default/files/documents/mooc_report-2013.pdf. Accessed 2015 Jul 3.

[9] Perna LW, Ruby A, Boruch RF, Wang N, Scull J, Ahmad S, et al Moving Through MOOCs: Understanding the Progression of Users in Massive Open Online Courses. Educ Res [Internet]. 2014 12 2;43(9):421–32. Available: http://edr.sagepub.com/content/early/2014/11/25/0013189X14562423.abstract. Accessed 2014 Dec 23.

[10] Wuensch KL, Aziz S, Ozan E, Kishore M, Tabrizi MHN. Pedagogical Characteristics of Online and Face-to-Face Classes. Proceedings from World Conference on E-learning in Corporate, Government, Healthcare, and Heather Education; 2008;7:523–32.

[11] McKinney D, Dyck JL, Luber ES. iTunes University and the classroom: Can podcasts replace Professors? Comput Educ [Internet]. 2009 Apr;52(3):617–23. Available: http://linkinghub.elsevier.com/retrieve/pii/S036013150800167X. Accessed 2014 Dec 4.

[12] Ferguson J, Tryjankowski AM. Online versus face‐to‐face learning: looking at modes of instruction in Master’s‐level courses. J Furth High Educ [Internet]. 2009 8 27;33(3):219–28. Available: http://www.tandfonline.com/doi/abs/10.1080/03098770903026149#.VZcKuEYYHaI. Accessed 2015 Jul 3.

[13] Xu D, Jaggars SS. The impact of online learning on students’ course outcomes: Evidence from a large community and technical college system. Econ Educ Rev [Internet]. 2013 12;37:46–57. Available: http://www.sciencedirect.com/science/article/pii/S0272775713001039. Accessed 2015 Apr 26.

[14] Unsworth N, McMillan BD, Brewer G a, Spillers GJ. Everyday attention failures: an individual differences investigation. J Exp Psychol Learn Mem Cogn [Internet]. 2012 11;38(6):1765–72. Available: http://www.ncbi.nlm.nih.gov/pubmed/22468805. Accessed 2015 Jan 5. 10.1037/a0028075 22468805

[15] Cameron P, Giuntoli D. Consciousness Sampling in the College Classroom or Is Anybody Listening?. Intellect [Internet]. 1971 11 30; Available: http://eric.ed.gov/?id=EJ065971. Accessed 2015 Jan 17.

[16] Lindquist SI, McLean JP. Daydreaming and its correlates in an educational environment. Learn Individ Differ [Internet]. 2011 4;21(2):158–67. Available: http://linkinghub.elsevier.com/retrieve/pii/S1041608011000045. Accessed 2014 Feb 5.

[17] Schoen JR. Use of consciousness sampling to study teaching methods. J Educ Res [Internet]. 1970;63(9):387–90. Available: http://www.jstor.org/discover/10.2307/27536016?sid=21105090228271&uid=3739600&uid=2&uid=3739256&uid=4. Accessed 2015 Jan 17.

[18] Risko EF, Anderson N, Sarwal A, Engelhardt M, Kingstone A. Everyday Attention: Variation in Mind Wandering and Memory in a Lecture. Appl Cogn Psychol [Internet]. 2012 3 25;26(2):234–42. Available: http://doi.wiley.com/10.1002/acp.1814. Accessed 2015 Jan 13.

[19] Szpunar KK, Khan NY, Schacter DL. Interpolated memory tests reduce mind wandering and improve learning of online lectures. Proc Natl Acad Sci U S A [Internet]. 2013 4 16; 110(16):6313–7. Available: http://www.pubmedcentral.nih.gov/articlerender.fcgi?artid=3631699&tool=pmcentrez&rendertype=abstract. Accessed 2014 Dec 14. 10.1073/pnas.1221764110 23576743 PMC3631699

[20] Risko EF, Buchanan D, Medimorec S, Kingstone A. Everyday attention: Mind wandering and computer use during lectures. Comput Educ [Internet]. 2013 10;68:275–83. Available: http://linkinghub.elsevier.com/retrieve/pii/S0360131513001218. Accessed 2015 Jan 7.

[21] Bond CF, Titus LJ. Social facilitation: A meta-analysis of 241 studies. Psychol Bull. 1983;94.2:265. 6356198

[22] Foulsham T, Cheng JT, Tracy JL, Henrich J, Kingstone A. Gaze allocation in a dynamic situation: effects of social status and speaking. Cognition [Internet]. 2010 12;117(3):319–31. Available: http://www.sciencedirect.com/science/article/pii/S0010027710002167. Accessed 2015 Jul 3. 10.1016/j.cognition.2010.09.003 20965502

[23] Huguet P, Galvaing MP, Monteil JM, Dumas F. Social presence effects in the Stroop task: Further evidence for an attentional view of social facilitation. J Pers Soc Psychol. 1999;77:1011 10573878 10.1037//0022-3514.77.5.1011

[24] Aragon S. Creating social presence in online environments In: Aragon S, editor. New Directions for Adult and Continuing Education. San Francisco: Jossey Bass; 2010 p.57–68.

[25] Laidlaw KEW, Foulsham T, Kuhn G, Kingstone A. Potential social interactions are important to social attention. Proc Natl Acad Sci U S A [Internet]. 2011 4 5;108(14):5548–53. Available: http://www.pnas.org/content/108/14/5548.short. Accessed 2015 Jul 3. 10.1073/pnas.1017022108 21436052 PMC3078350

[26] Richardson JC. PD, Swan KPD. Examing Social Presence in Online Courses in Relation to Students’ Perceived Learning and Satisfaction. 2003 Feb 1; Available: https://www.ideals.illinois.edu/handle/2142/18713. Accessed 2015 Jul 3.

[27] Kingstone A, Smilek D, Eastwood JD. Cognitive Ethology: a new approach for studying human cognition. Br J Psychol. 2008;99(Pt 3):317–40. 17977481 10.1348/000712607X251243

[28] Forster S, Lavie N. Distracted by your mind? Individual differences in distractibility predict mind wandering. J Exp Psychol Learn Mem Cogn [Internet]. 2014;40(1):251–60. Available: http://www.ncbi.nlm.nih.gov/pubmed/23957365 10.1037/a0034108 23957365

[29] Giambra LM, Grodsky A. Task-Unrelated Images and Thoughts While Reading Shorr JE, Robin P, Connella JA, Wolpin M, editors. Imagery: Current Perspective. Boston, MA: Springer US; 1989 p. 27–31.

[30] Unsworth N, McMillan BD. Mind wandering and reading comprehension: examining the roles of working memory capacity, interest, motivation, and topic experience. J Exp Psychol Learn Mem Cogn [Internet]. 2013 5;39(3):832–42. Available: http://www.ncbi.nlm.nih.gov/pubmed/22905931. Accessed 2015 Jan 9. 10.1037/a0029669 22905931

[31] Garrison DR. E-Learning in the 21st Century: A Framework for Research and Practice [Internet]. Taylor & Francis; 2011 184 p. Available: https://books.google.com/books?hl=en&lr=&id=aodjWyjxYbYC&pgis=1. Accessed 2015 Jul 3.

[32] Erichsen EA, Bolliger DU. Towards understanding international graduate student isolation in traditional and online environments. Educ Technol Res Dev. 2010 7 3;59(3):309–26.

[33] Sung E, Mayer RE. Five facets of social presence in online distance education. Comput Human Behav [Internet]. 2012 9;28(5):1738–47. Available: http://linkinghub.elsevier.com/retrieve/pii/S0747563212001185. Accessed 2015 Jan 8.

[34] Meyer R. What It’s Like to Teach a MOOC (and What the Heck's a MOOC?)—The Atlantic [Internet]. 2012. Available: http://www.theatlantic.com/technology/archive/2012/07/what-its-like-to-teach-a-mooc-and-what-the-hecks-a-mooc/260000/. Accessed 2015 Jul 5.

[35] Onah DFO, Sinclair J, Boyatt R. Dropout rates of massive open online courses : behavioural patterns [Internet]. EDULEARN14 Proceedings. IATED Academy; 2014. Available: http://wrap.warwick.ac.uk/65543/1/WRAP_9770711-cs-070115-edulearn2014.pdf. Accessed 2015 Jul 3.

[36] Pomerantz J. Data about the Metadata MOOC round 2 [Internet]. 2014. Available: http://jeffrey.pomerantz.name/2014/10/data-about-the-metadata-mooc-round-2/. Accessed 2015 Jul 5.

