# Supplementary material for: Memory for Lectures: How Lecture Format Impacts the Learning Experience
Source: PLoS One. 2015 Nov 11;10(11):e0141587. doi: 10.1371/journal.pone.0141587 (PMC4641615; doi:10.1371/journal.pone.0141587)
Supplement: S1 Appendix — (DOCX) [file pone.0141587.s001.docx]

**Appendix A: Memory test Questions**

Psychotherapy Questions

1. In the 19^th^ century, Philippe Pinel lobbied for humane asylums in the US. (False)
2. Psychoanalytic theory focuses on working through conscious wishes and feelings. (False)
3. Humanistic therapy focuses providing on empathy as an important technique in aiding patients. (True)
4. Compulsive behaviors are learned through classical conditioning. (False)
5. Cognitive behavioral therapy is shorter than psychoanalysis. (True)
6. Humanistic approach is the best approach to treat low self-esteem. (True)

Drug Therapy Questions

1. Systemic therapy is a form of biomedical treatment. (False)
2. Serotonin is an excitatory neurotransmitter. (False)
3. Prozac is an SSRI (Selective Serotonin Reuptake Inhibitor). (True)
4. Antianxiety drugs are slower to take effect than antidepressants. (False)
5. Antipsychotic drugs increase dopamine in the synapses. (False)
6. Psychiatrists, but not clinical psychologists, can prescribe drugs. (True)
