# Supplementary figures and images for: Memory for Lectures: How Lecture Format Impacts the Learning Experience
Source: PLoS One. 2015 Nov 11;10(11):e0141587. doi: 10.1371/journal.pone.0141587 (PMC4641615; doi:10.1371/journal.pone.0141587)

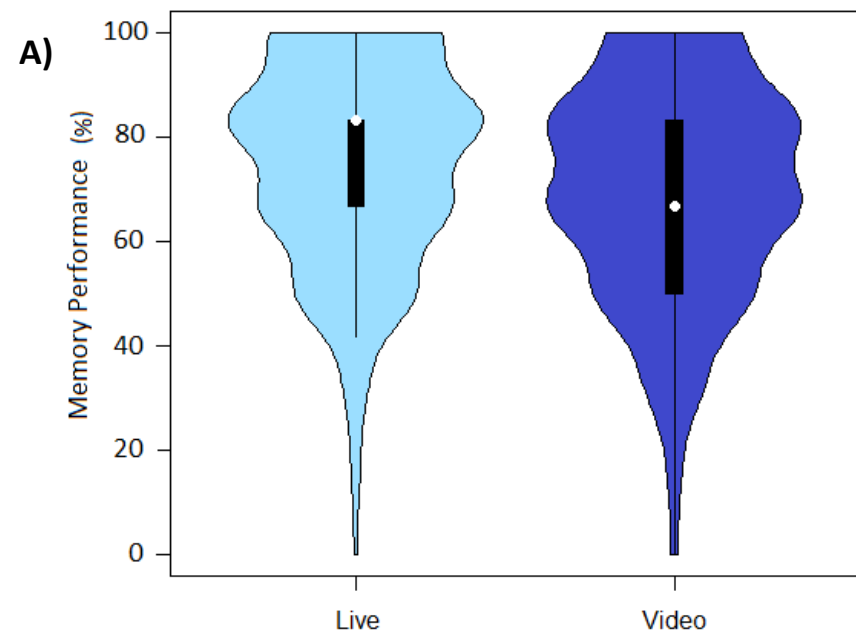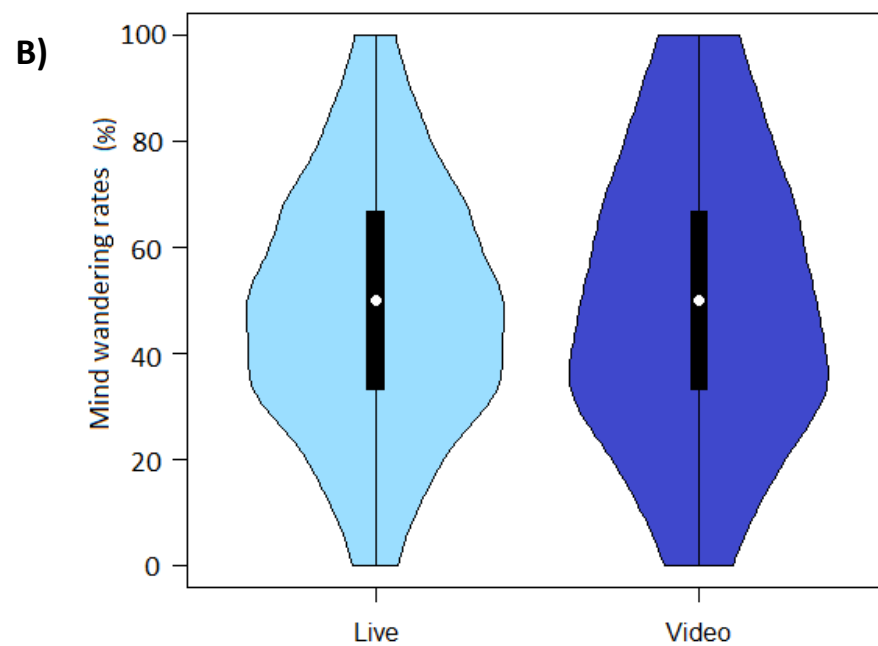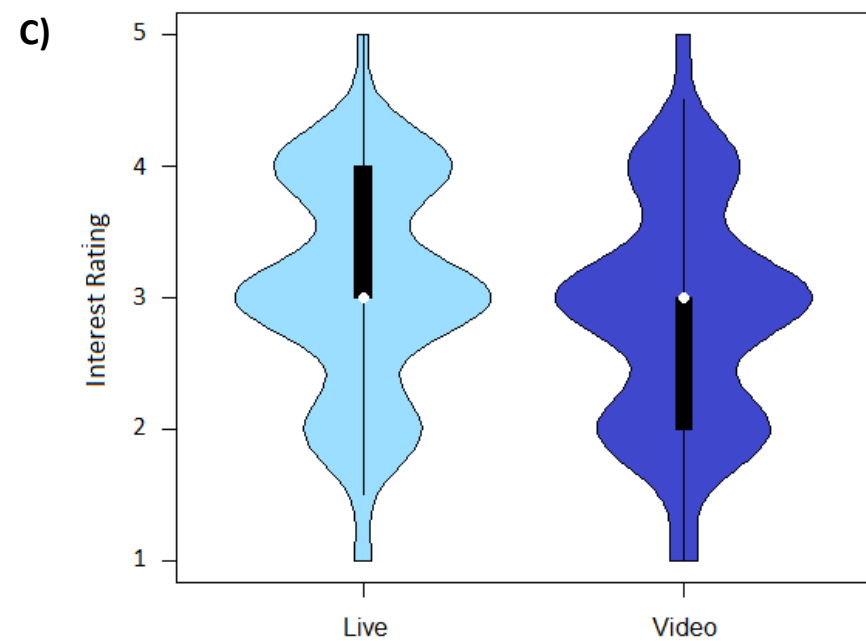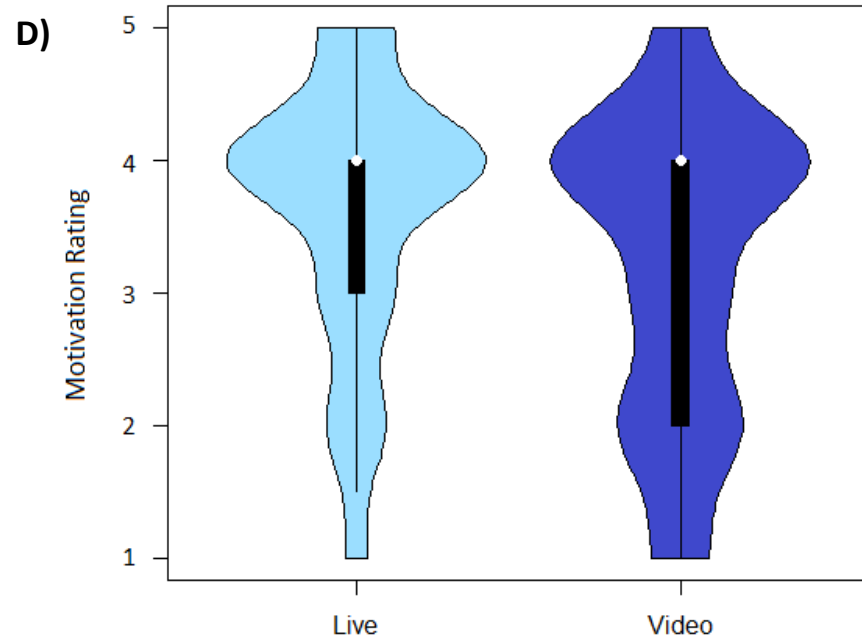

Supplement: S1 Fig — Violin plots indicate the density distribution each dependent variable. Light coloured plots represent performance in the Live condition and Dark coloured plots represent performance in the Video condition. The white dot represents the median and the black bar represents the interquartile range. (A) Memory performance percentage. (B) Percentage of mind wandering reports. (C) Interest Ratings, where 5 indicated high interest and 1 indicates low interest. (D) Motivation Ratings, where 5 indicated high motivation to attend and 1 indicates low motivation to attend. (PDF) [file pone.0141587.s002.pdf]

A)

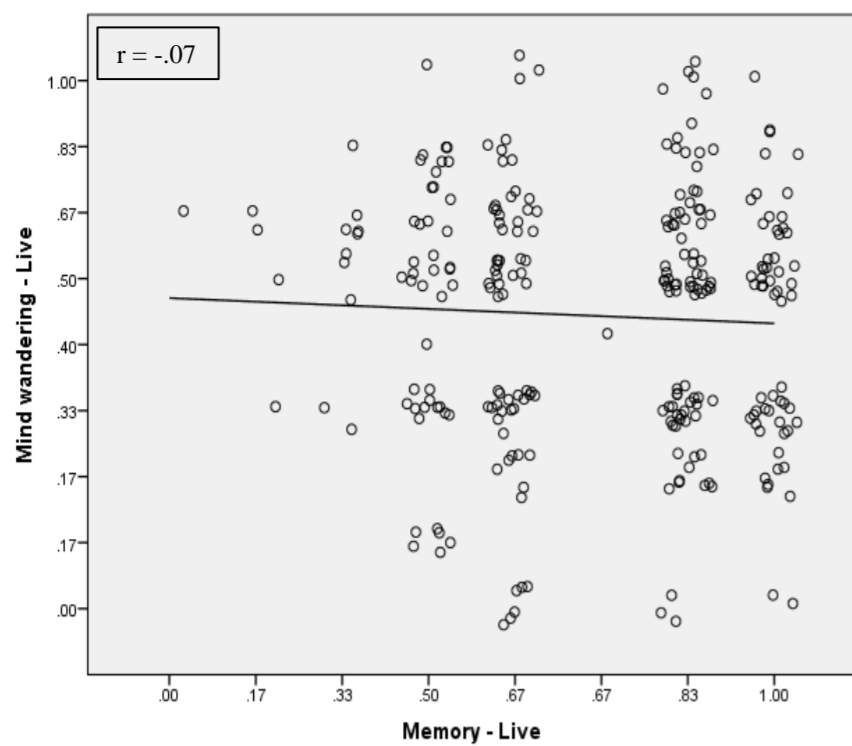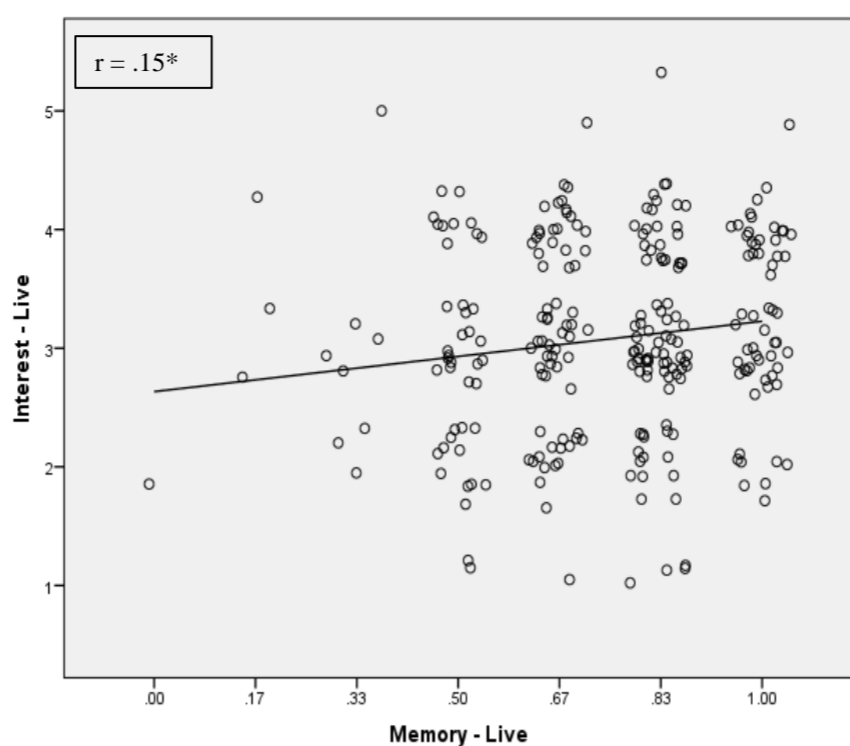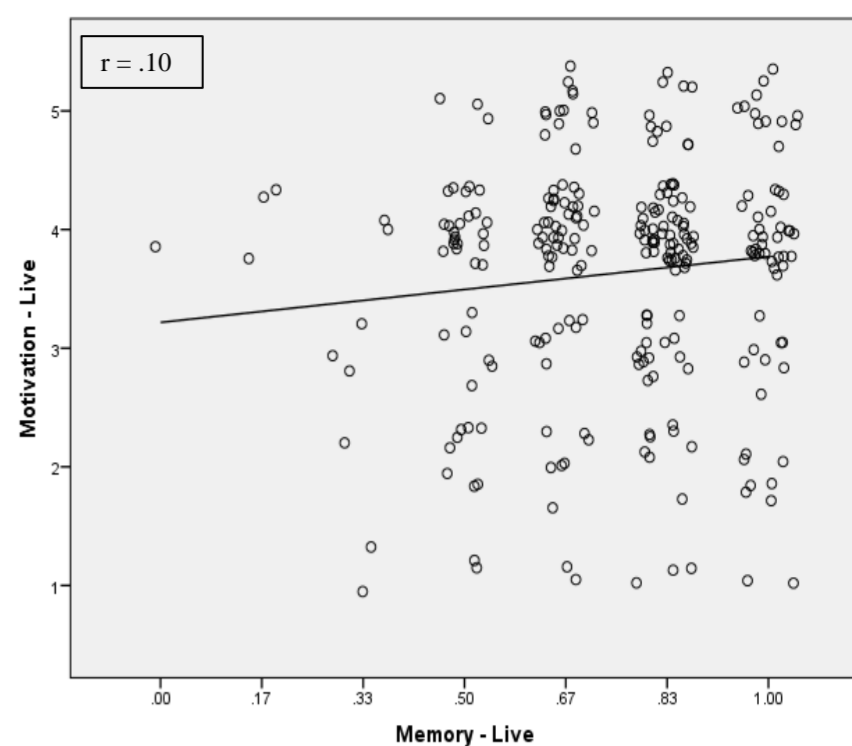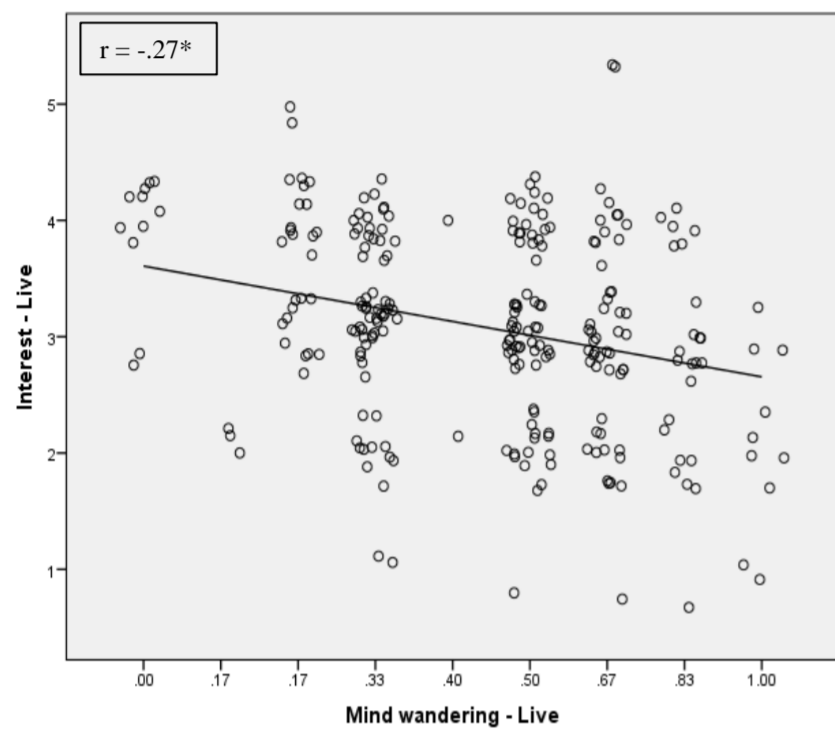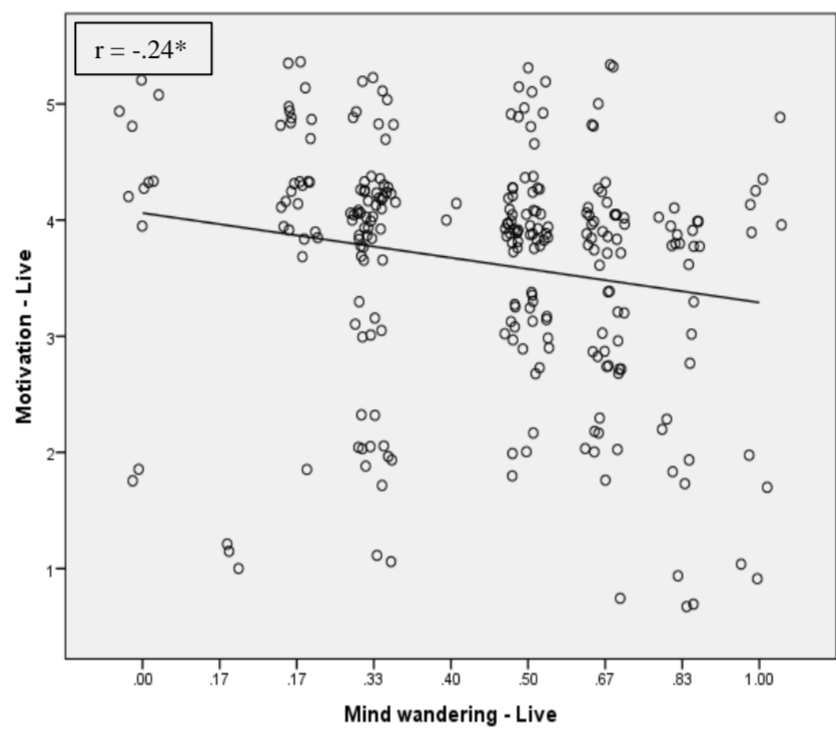

B)

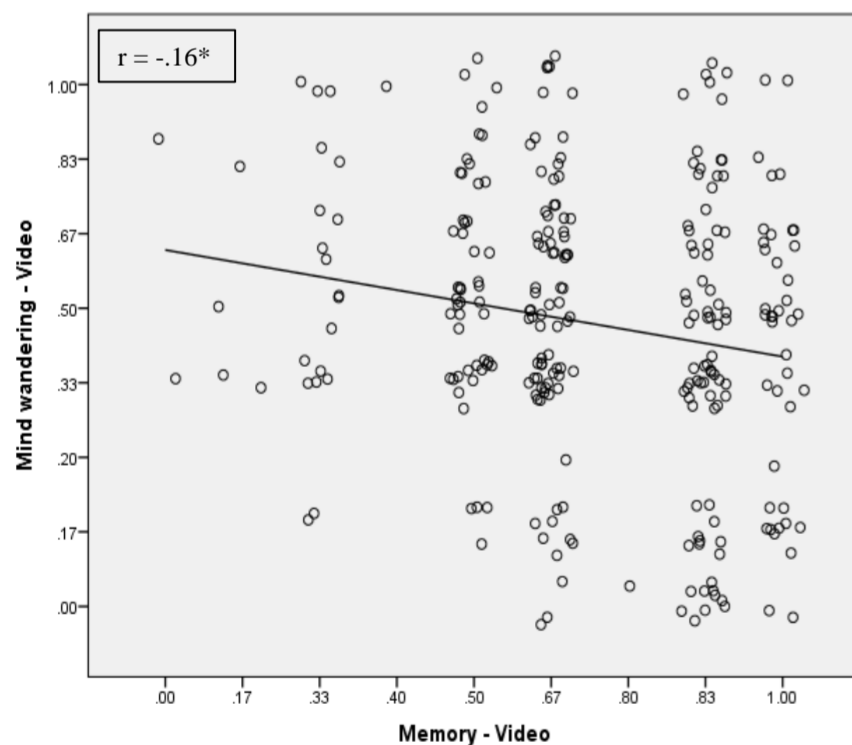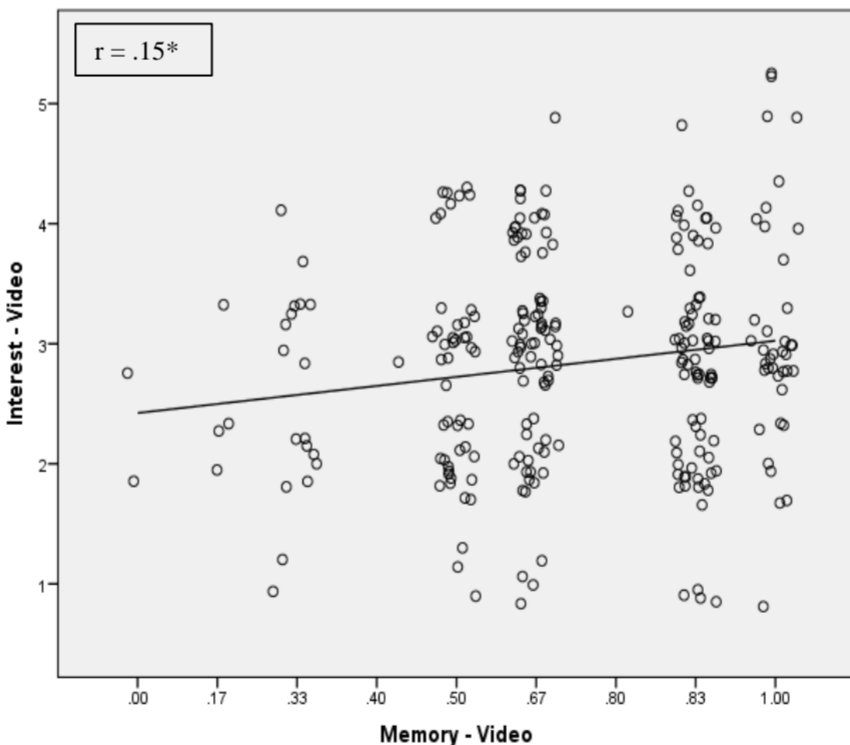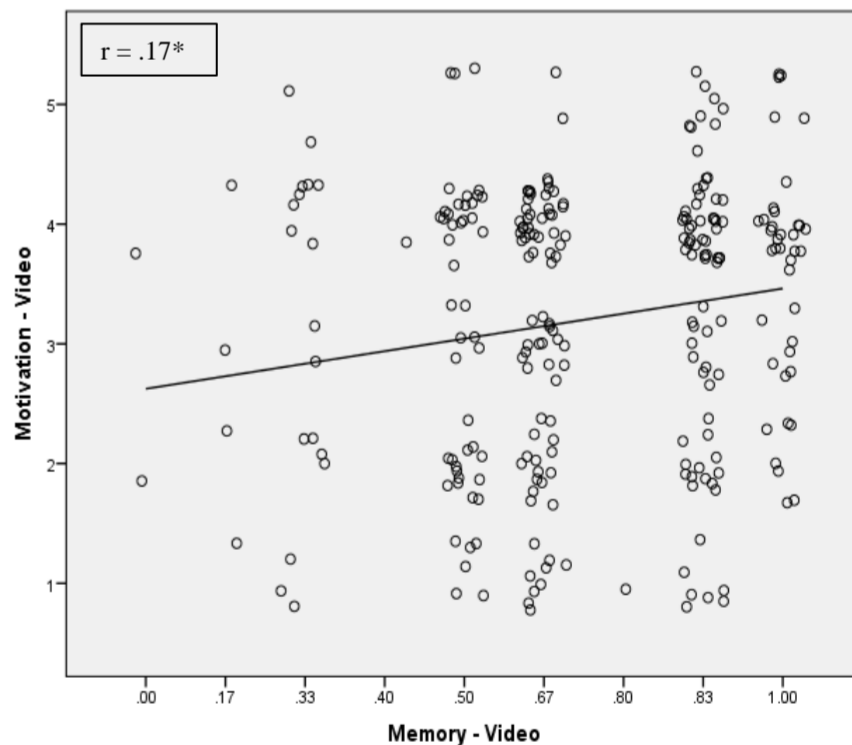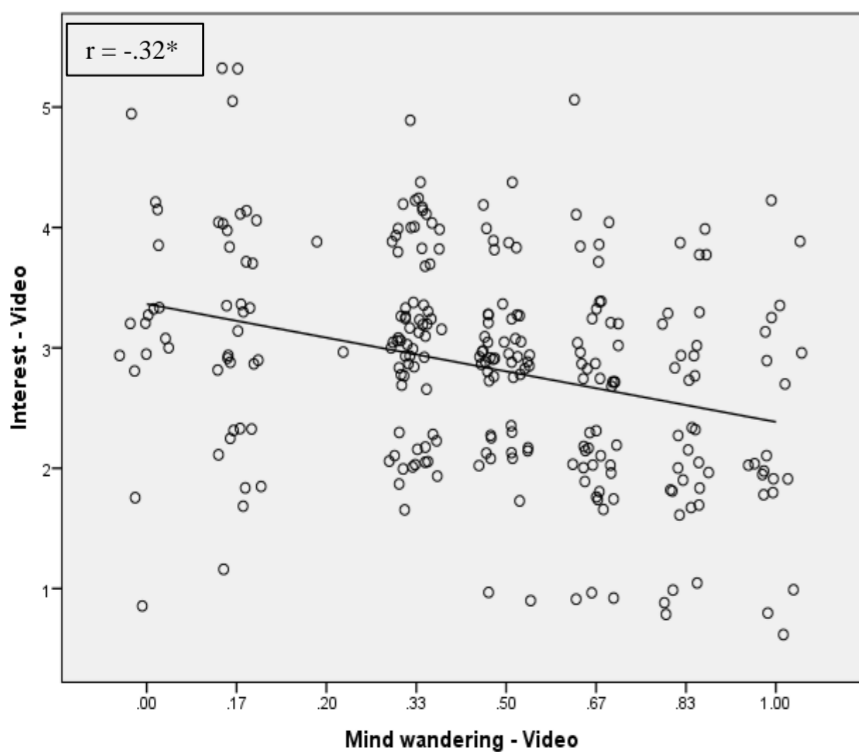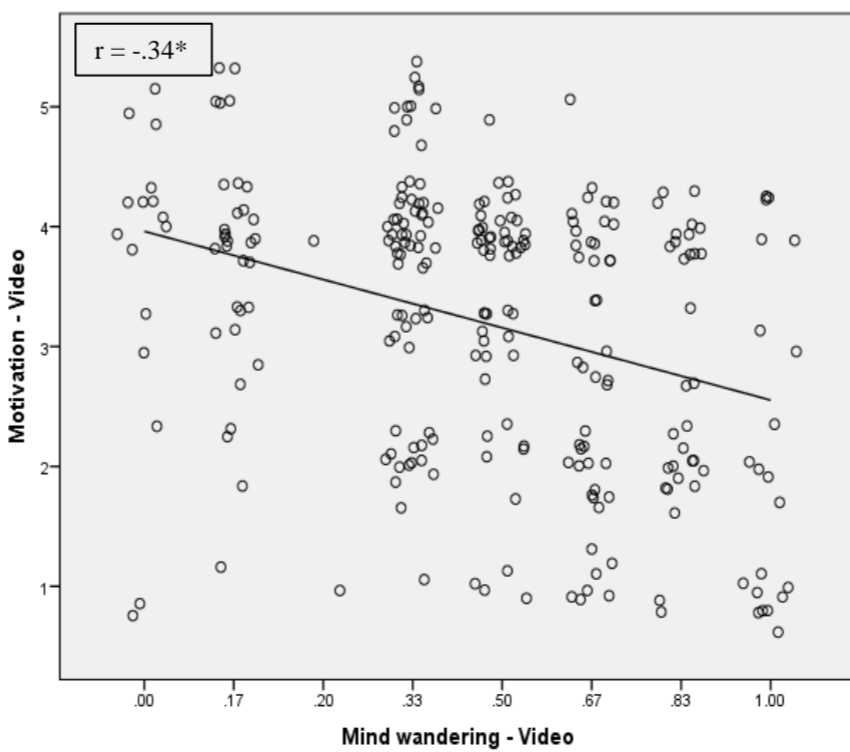

Supplement: S2 Fig — Jittering was used to distinctly display data points that would otherwise overlap (due to scales not being continuous) and provide a clear visualization of the data. (A) Displays correlations between the variables for the Live condition. (B) Displays correlations between the variables for the Video condition. (PDF) [file pone.0141587.s003.pdf]
